# Effects of Xenobiotic Compounds on Preeclampsia and Potential Mechanisms

**DOI:** 10.3390/toxics11060492

**Published:** 2023-05-31

**Authors:** Miaoliang Wu, Fuhui Yan, Qian Liu, Ganzhong Liao, Yilin Shen, Zhi Bai, Xiaoshan Liu

**Affiliations:** 1 The First Dongguan Affiliated Hospital, Guangdong Medical University, Dongguan 523808, China; 2 School of Public Health, Dongguan Key Laboratory of Environmental Medicine, Guangdong Medical University, Dongguan 523808, Chinam13192852105@163.com (Y.S.)

**Keywords:** endocrine disruptor, placenta, preeclampsia

## Abstract

Preeclampsia (PE) refers to a disease with new hypertension and albuminuria or other end-organ damage after 20 weeks of pregnancy. As a major complication of pregnancy, PE can increase the morbidity and mortality of pregnant women and fetuses and cause serious social burden. Recently, it has been found that exposure to xenobiotic compounds, especially endocrine disruptors in the environment, may contribute to the development of PE. However, the underlying mechanism is still unclear. It is generally believed that PE is related to placental dysplasia, spiral artery remodelling failure, oxidative stress, etc. Therefore, in order to better prevent the occurrence of PE and reduce the damage and impact on mother and fetus, this paper reviews the role and potential mechanism of PE induced by exogenous chemicals and provides an outlook on the environmental etiology of PE.

## 1. Conception of Preeclampsia

Preeclampsia (PE) is a multi-organ, multi-system syndrome specific to pregnancy and postpartum emergency with systolic blood pressure above 140 mmHg and/or diastolic blood pressure above 90 mmHg [1,2]. PE patients have at least one of the following syndromes: proteinuria, maternal organ dysfunction (e.g., liver and kidney damage), or uteroplacental dysfunction (e.g., fetal growth restriction, stillbirth) [3]. It is a hypertensive disorder in pregnancy, and the prevalence of PE is as high as 2–8% in the world [4]. A cross-sectional study showed similar prevalence of PE in Sweden (2.8%) and China (2.2%), but two-thirds of cases in Sweden were mild and two-thirds in China were severe, and the mortality rate of pregnant women in China was almost 10 times higher [5]. The prevalence of PE in developing countries is much higher than that in developed countries, with the prevalence of PE being only 3–8% in the United States, compared with 28% in India [6,7]. In severe cases, PE can lead to fetal growth restriction, placental abruption, premature birth, and stillbirth. Even after recovery, pregnant women are at higher risk of long-term complications such as hypertension, stroke, heart failure, kidney disease, and Alzheimer’s disease, with a subsequent increased risk of cardiovascular disease in their offspring or PE during pregnancy [8,9].

### 1.1. Etiology of PE

The etiology of PE remains unclear. Studies have shown that genetic material [10], maternal health status [11], lifestyle [12], nutrient intake [13], exogenous compound exposure, [14] and other factors may increase the risk of PE. Ferreira et al. found that the mutation of ERAP2 has a genetic association with the PE [10]. The genetic rate of pregnant women with family history of PE is 55%, and the risk of maternal and fetal genetic contributions to PE is 30–35% and 20%, respectively [15]. The study by Bartsch et al. showed that several clinical risk factors, including maternal antiphospholipid antibody syndrome, chronic hypertension, pregestational diabetes, pregnancy body mass index, and use of assisted reproductive technology are prominent risk factors of PE [11]. Prevention of these clinical risk factors will significantly reduce the risk of early occurrence of PE. Phipps et al. found that the maternal smoking or long-term sexual cohabitation are risk factors for PE [12]. There is also a certain connection between a mother’s nutrient intake and the occurrence of PE, such as vitamin D, calcium. Mirzakhani et al. investigated the association between maternal vitamin D intake and the occurrence of PE, and found that maternal vitamin D deficiency in early pregnancy was a risk factor for PE [13]. Duvekot et al. conducted a case-control study, comparing milk intake between PE patients and normal women, and showed that lower milk intake, resulting in reduced calcium intake, increased the risk of PE [16]. In addition, exposure to toxic and harmful chemicals during pregnancy can lead to adverse pregnancy outcomes such as PE. A cohort study incorporating 90 female workers showed that occupational exposure to organic solvents, such as aldehydes, significantly increased the occurrence of PE [14]. Dadvand et al. used a logistic regression model to evaluate the correlation between occurrence of PE and prenatal particulate matter exposure, and found that exposure to particles increased the risk of PE; for each unit of exposure of PM10 brake dust, the risk of PE increased by 44% [17]. In this paper, we review the research progress and potential mechanisms of PE induced by exogenous pollutants in recent years, so as to provide a evidence for the risk assessment of these pollutants.

### 1.2. Pathogenesis of PE

**Figure 1:**
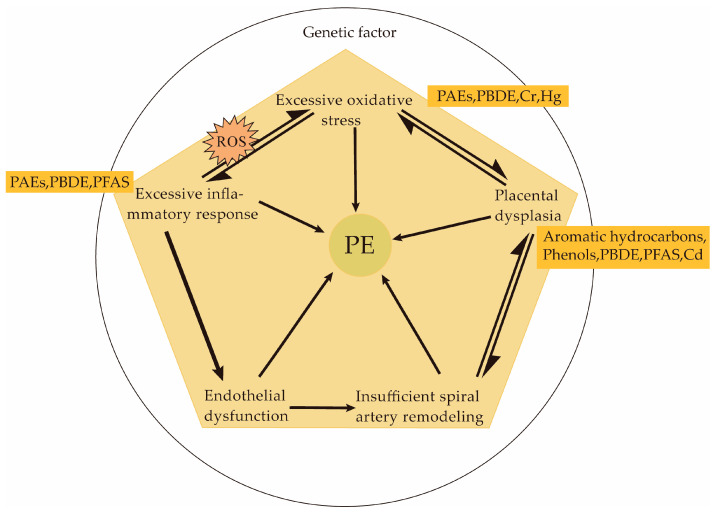
Pathogenesis of PE and potential mechanism of exogenous compounds.

The pathogenesis of PE is not fully understood, but some progress has been made in recent decades. The most widely accepted is the two-stage model [18,19,20,21]: (i) subclinical stage—in early pregnancy, the placenta shallowly settles due to insufficient invasion, resulting in insufficient remodelling of the uterine spiral artery and placental ischemia and hypoxia; (ii) clinical stage—The anti-angiogenic factors released are by the ischemic placenta into maternal circulation, leading to vascular endothelial dysfunction. As shown in Figure 1, currently, various genetics, structural, and angiogenic factors have been implicated in PE, including spiral artery remodelling, placental redox, and immune tolerance at the fetal–maternal interface, and the balance of anti-angiogenic and angiogenic factors. 

#### 1.2.1. Insufficient Spiral Artery Remodelling

During embryo implantation in normal pregnancy, placental trophoblasts invade the maternal uterus to a certain depth to remodel the uterine spiral arteries into low-resistance vessels, thereby optimizing maternal–fetal blood flow, known as spiral artery remodelling [22]. During the remodelling process, extravillous trophoblasts (EVTs), which are invasive cells differentiated from cytotrophoblast cells (CTBs), invade the decidua and anchor the placenta to the uterus, replacing some of the endothelial cells in the vessel wall itself to increase the diameter of the spiral artery by 5–10 times, resulting in a “high flow, low resistance” remodeled vessel [23]. The main function of this process is to nourish the uterine basement membrane and provide conditions for fetal development [24]. The remodelling of the Spiral artery mainly includes two main processes: metaplasia-related and trophoblast-related remodelling [25]. Therefore, defective metaplasia and insufficient EVT invasion are two reasons for the failure of spiral artery remodelling. In patients with PE, EVT infiltration in early gestation only reaches the metaplastic layer without significant invasion of the muscular layer, leading to failure of spiral artery remodelling and, consequently, to inadequate placental perfusion [26].

#### 1.2.2. Oxidative Stress

There is a balance between oxidation and antioxidation in the human body. When the anoxia/reoxygenation transition mechanism of the placenta is abnormal, trophoblast cells are in a continuous hypoxic microenvironment and undergo oxidative stress [27]. Interestingly, increased placental mitochondrial activity and ROS production during normal early pregnancy can cause placental oxidative stress [19]. ROS generated by oxidative stress can increase the catabolism of vasodilator nitric oxide (NO), thus causing arteriolar spasm, and further ischemia of the placental tissue exacerbates oxidative stress [28]. The chain reaction could lead to an increase in anti-angiogenic factors, vascular intimal injury, and then trigger a series of pathophysiological processes such as endothelial dysfunction. In recent years, researchers have found that placental oxidative stress can also lead to reduced release of extracellular vesicles (EV), which play an important role in regulating endometrial embryo implantation, EVT invasion, maternal immune response, and spiral artery remodelling [29].

#### 1.2.3. Excessive Inflammatory Response

Systemic inflammation is a physiological characteristic of normal pregnancy [30,31]. The inflammatory response is over-activated and progressively increased with an increase in pro-inflammatory cytokines and anti-angiogenic factors in the intrauterine environment and in the maternal circulation, which is an important cause of PE [32]. Studies have shown that the number and proportion of immune cells in PE patients are altered [31]. An increased ratio of pro-inflammatory cytokines to anti-inflammatory cytokines in PE patients had been observed [33]. Widespread dysregulation of the immune system will accelerate the production of pro-inflammatory cytokines, and the complement system (classical and lectin complement pathways) will be over-activated as part of the compensatory mechanism, leading to greater terminal activation and excessive inflammation. Meanwhile, inflammatory response and oxidative stress have cross-talk. Excessive ROS generated by oxidative stress can activate inflammasome NLRP3 and release inflammatory factors, resulting in subsequent inflammatory cascade and cell damage [34].

#### 1.2.4. Endothelial Dysfunction

Endothelial cells cover the inner lining of blood vessels and form a barrier between blood and the extravascular matrix to maintain transport of solutes, fluids, and cells [34]. Imbalance of angiogenic factors is an important cause of endothelial dysfunction in PE patients. Vascular endothelial growth factor (VEGF) and placental growth factor (PIGF) are the most important angiogenic factors in normal pregnant maternal serum. VEGF can not only promote the proliferation of endothelial cells and maintain the activity of endothelial cells, but also promote the release of NO and diastolic prostaglandin, dilate blood vessels, and increase vascular permeability [35,36]. PIGF can increase the biological activity of VEGF, relax blood vessels, and prompt angiogenesis [37]. Physiological dysfunction of the endothelium promotes Endothelin-1 (ET-1) production, which further induces hypertension and proteinuria and inhibits the release of renin. Increased blood pressure also inhibits renin and aldosterone activity. Moreover, decreased activation of the renin-angiotensin-aldosterone system (RAAS) leads to a decrease in circulating blood volume, further reducing placental perfusion [26]. Both poor perfusion and excess soluble vascular endothelial growth factor receptor 1 (sFlt-1) can lead to increased endothelial dysfunction [38]. In addition, sFlt-1 has also been shown to competitively bind VEGF and PIGF, resulting in impaired endothelial relaxation and reduced angiogenesis [39,40].

#### 1.2.5. Genetic Factor

The inflammation-related genes INHBA, OPKR1, and TPBG are considered as genetic biomarkers of PE. The up-regulation of INHBA promotes human trophoblast invasion and plays an important role in early embryo implantation. The specific mechanism of OPKR1 in PE remains to be studied, but it may be related to the production of ROS. TPBG expression is increased in the placenta of PE patients, which may be related to epithelial–mesenchymal transition (EMT) during placentation [31]. mRNA LIF can promote trophoblast migration and invasion, but its targeted regulation by miRNAs results in reduced embryo implantation [41]. Placentas from PE have increased miRNA-155 and decreased endothelial nitric oxide synthase (eNOS) expression [42].

## 2. Role and Mechanism of PE Induction by Exogenous Compounds

### 2.1. Phenols

Exposure to phenols have been associated with PE. Bisphenol A (BPA) has been studied extensively. BPA is widely used in the manufacture of epoxy resins and polycarbonate polymers. It is found in a wide range of consumer products such as water bottles, metal coatings, flooring, and thermal paper [43]. BPA is readily released from these products, and the main route of human exposure is through diet [44]. A case–control study showed that maternal serum BPA concentration was positively correlated with the incidence of PE, with adjusted OR = 16.46 [43]. In a prenatal mouse BPA exposure test, Tachibana et al. found that BPA impaired placentation in mice, degenerated both trophoblast giant cells and the spongiotrophoblast layer, decreased the proportion of the labyrinthine zone per the total placenta, and resulted in abortion or neonatal mortality [45]. Imanishi et al. also showed that BPA affected the mRNA expression of nine nuclear receptors in the placenta and, thus, disrupted placental function [46]. Tait et al. exposed CD-1 mice in early pregnancy to 0.5 mg/kg and 50 mg/kg BPA and found that BPA narrowed maternal vessels in the low-dose treatment group, inhibited blood vessel development and branching, and irregularly dilated the maternal vessels in the labyrinth in the high-dose group, suggesting that the placenta is an important target of BPA [47]. Ye et al. found that BPA could induce PE in the intrauterine exposure model of CD-1 mice, alter expression of invasion-related genes and inhibit cell invasion in HTR-8/SVneo cells [48]. Cantonwine et al. showed that BPA can affect the proliferation of trophoblast cells through estrogen-related receptor γ (ERRγ1) [49]. Benachour et al. also showed that BPA induced tumor necrosis factor (TNFα)-mediated apoptosis of human primary trophoblast cells (CTB), resulting in insufficient trophoblasts invasion [50]. BPS, as a substitute for BPA, can also interfere with trophoblast cell fusion in the sheep placenta [51]. In addition, BPA exposure increases the biomarker sFlt-1/PIGF, which is associated with angiogenesis during pregnancy [52].

### 2.2. Aromatic Hydrocarbon

Benzopyrene (BaP) is one of the polycyclic aromatic hydrocarbons (PAHs) found in the environment, mainly derived from volcanic eruptions, vehicle exhaust, and the incomplete combustion of wood, coal, and cigarette smoke. Humans are exposed to BaP via oral, respiratory, and dermal contact [53]. In a case–control study, Wu et al. collected and detected BaP DNA adducts in blood and abortion tissues, and found that the risk of early abortion was increased in mothers exposed to high levels of BaP, with adjusted OR = 4.27 [54]. Li et al. exposed mice to BaP and found that BaP can impair endometrial receptivity and inhibit decidualisation and decidual angiogenesis in early pregnancy [53]. BaP also inhibits trophoblast cell migration and invasion, resulting in inadequate remodelling of the spiral arteries [55]. Wang et al. found that BenzoA-Pyrene-Diol-Epoxide (BPDE), one of the metabolites of BaP, also significantly inhibited the trophoblast cell migration and invasion [56,57]. These data suggest that maternal exposure to BaP during pregnancy may inhibit the migration and invasion of trophoblast cells, providing the basis for the development of PE.

### 2.3. Phthalates

Phthalates (PAEs) are common plasticisers that are widely used in personal care products and food packaging industries [58]. PAEs can easily migrate into various environmental media during storage or use, and thus enter the human body through respiratory, digestive, and dermal contact [59]. A prospective cohort study found that several metabolites of PAEs were significantly positively correlated with the increase in hypertensive syndrome in pregnancy and systolic blood pressure of pregnancy [58]. Deliveries with PE or intrauterine growth restriction had a slightly higher odds ratio for DEHP metabolites in the first trimester, with OR = 1.33 [60]. These studies all suggest a strong association between PAEs and increased risk of PE. Zong et al. found that exposure of pregnant mice to DEHP disrupted vascularisation of the placental labyrinth zone, inhibited placental development, and reduced embryo implantation [61]. In addition, exposure to mono-2-ethylhexyl phthalate (MEHP), a metabolite of DEHP, was found to over-activate oxidative stress, leading to an increase in ROS and oxidative damage to DNA [62]. Similarly, Meruvu et al. showed that MEHP up-regulated the expression of oxidative stress-responsive miR17-5p, miR-155-5p, and miR-126-3p in HTR8/SVneo cells in a dose-and-time-dependent manner [63]. Shoaito et al. also found that MEHP might enhance the sensitivity of intracellular oxidative stress by activating the mitogen-activated protein kinase (MAPK) signalling pathway in trophoblasts [64]. Meanwhile, a study also showed that MEHP exposure significantly inhibited the activity of MMP-9, upregulated the expression of the tissue inhibitor matrix metalloproteinase-1 (TIMP-1), and inhibited trophoblast invasion via PPARγ [65].

### 2.4. Flame Retardants

Polybrominated diphenyl ether congeners (PBDEs) are widely used flame retardants in household appliances, plastics, televisions, curtains, and upholstery, and humans are exposed to PBDEs through inhalation and ingestion [66]. Among them, polybrominated diphenyl ether (BDE-47) has been found at the highest level. Varshavsky et al. and Kwon et al. measured the BDE-47 levels in the placenta and maternal serum during the second trimester of pregnancy, and found that the concentration of BDE-47 was correlated with altered levels of vascular endothelial VE-cadherin, which would affect the invasive ability of trophoblasts [67,68]. In addition, BDE-47 may affect placental function, which was associated with oxidative stress, pro-inflammatory mediators, and decreased monocyte and trophoblast viability [67,69]. Wang et al. demonstrated that triphenyl phosphate (TPhP), one of the organophosphate flame retardants, can activate PPARγ in JEG-3 trophoblast cells, interfere with hormone secretion, and induce endoplasmic reticulum stress and cell apoptosis [70]. Hong et al. showed that TPhP could accumulate in the placenta and activate placental PPARγ to impair pregnancy outcome [71]. 

### 2.5. Pesticides

Pesticides, including insecticides, fungicides, herbicides, and plant growth regulators, are widely used in agriculture, households and for vector control, among which insecticides and herbicides have adverse effects on ecosystems and human health [72]. So far, research on the association between pesticide exposure and the occurrence of PE is limited, and the results are inconsistent. For example, Saldana et al. analysed self-reported data from 11,274 farm women from 1993 to 1997 and found that women who engaged in activities with possible pesticide exposure during the first trimester of pregnancy may have an increased risk of PE [73]. Toichuev et al. measured organochlorine pesticides (OCPs) in 240 placentae and found that OCPs-exposed mothers had a high risk of PE, and COPs-exposed newborns had a high risk of low birth weight, stillbirth, and congenital malformations [74]. Murray et al. suggested that exposure to dichlorodiphenyltrichloroethane (DDT) and dichlorodiphenyl dichloroethylene (DDE) may increase the risk of developing pregnancy-induced hypertension [75]. However, Savitz et al. examined OCPs in serum and found virtually no association between the level of DDE and the risk of PE, while DDT was negatively associated with the risk of pregnancy-induced hypertension [76]. Saunders et al. conducted a prospective mother–infant cohort study, measured the plasma levels of chlordecone (one of the OCPs) in 779 pregnant women, and found no significant association between chlordecone exposure and the risk of PE [77]. 

Due to the persistent adverse effects of OCPs on human health and the environment, organophosphorus compounds (OPPs) have become the most widely used pesticides. Warembourg et al. measured the urinary levels of OPPs and found no association between OPPs exposure and the risk of PE, possibly due to the limited population sample size [78]. Although environmental epidemiological studies have not shown a significant association between prenatal pesticide exposure and the risk of PE, in vivo toxicological studies and in vitro mechanistic data suggested the potential toxicity of pesticides on PE [79]. Levario-Carrillo et al. exposed pregnant rats to methyl parathion (MP) and showed vascular congestion in the placental labyrinth layer and fibrosis-like changes in the decidua [80]. Chlorpyrifos (CPF) is also one of the most widely used OPPs in the world. Some vitro experiments have shown that CPF can induce apoptosis of JAR cells, alter the expression of genes associated with placental function, disrupt redox balance, and trigger antioxidant defense mechanisms as well as ER stress in JEG-3 [81]. In addition, Triclosan (TCS) is a broad spectrum of synthetic antibacterial agents commonly used in soaps, hand sanitizers, toothpastes, cosmetics, antiperspirants, bedding, and food products. Humans are frequently exposed to this compound through oral and dermal contact [82]. Exposure to TCS in pregnant mice resulted in a significant reduction in placental diameter and fetal body weight [82,83]; exposure to TCS in pregnant rats also resulted in reducing uterine weight and an increased abortion rate. The bioaccumulation of TCS in the placenta suggested that the placenta may be the target tissue of TCS [84]. In vitro studies also investigated the potential molecular mechanisms. One study showed that TCS disturbed steroidogenesis in JEG-3 cells and increased levels of progesterone and estradiol, but decreased hCG secretion [85]. TCS down-regulated PPARγ and its downstream angiogenesis-related gene expression, but up-regulated inflammatory gene expression, and inhibited cell migration and angiogenesis in HTR-8/SVneo cells and JEG-3 cells [86].

### 2.6. Per- and Polyfluoroalkyl Substances

Per- and polyfluoroalkyl substances (PFAS) with good molecular stability, hydrophobicity and lipophobicity are commonly used in nonstick cookware, water repellant clothing, cosmetic products, paper coatings, and fire-fighting foams [87]. Humans are exposed to PFAS via multiple routes, with drinking water ingestion being the major route. Wikström et al. conducted a prospective cohort study and found that the concentration of perfluorooctane sulfonates (PFOS) in the serum of early pregnancy were positively associated with the risk of PE, OR> 1 [88]. In vivo studies have shown that exposure to PFAS alters placental weight, disrupts its labyrinthine architecture, and induces congestion and clot formation [89]. Szilagyi et al. described that PFAS can affect placental development by disrupting the balance of PPAR-α and PPAR-γ in trophoblast cells to induce pregnancy complications, such as PE and gestational hypertension [90]. Blake et al. evaluated the toxicity of 42 PFAS on human choriocarcinoma cell JEG-3 and found that PFOS, perfluorooctanoic acid (PFOA), and ammonium perfluoro-2-methyl-3-oxahexanoate (GenX) interfered with the gene expression of oxidative stress-related genes GPEX1, GPER1, SOD1, and ABCG2, and inhibited cell migration [91]. Szilagyi et al. showed that PFOS, PFOA, and GenX could reduce the cytokine CCL2 and receptor CCR4 and inhibit cell migration and invasion [92].

### 2.7. Others

In addition to the exogenous compounds mentioned above, exposure to a variety of heavy metals has also been correlated with the development of PE. Liu et al. and Li et al. found that the level of cadmium (Cd) and the risk of PE were positively correlated, i.e., elevated Cd levels increased the risk of PE [93,94]. Zhang et al. used cadmium chloride (CdCl_2_) to develop a new model of PE in rats, which showed cardinal symptom of PE such as hypertension, proteinuria, placental abnormalities, and fetal weight loss [95]. Brooks et al. also found that exposure of JEG-3 cells to Cd increased the expression of the TGF-β and hindered placental trophoblast migration [96]. In addition to Cd, Wang et al. found that chromium (Cr), mercury (Hg), lead (Pb), and arsenic (As) were positively correlated with the occurrence of PE in an epidemiological study in Taiyuan City, China [97]. Maduray et al. conducted a cross-sectional study and found significantly higher Cr concentrations in hair samples from the PE group than from the control group [98]. In addition, epidemiological studies have also shown that environmental exposure to Pb, antimony (Sb), and manganese (Mn) increases the risk of PE [99]. Zahran et al. found in their study that the probability of PE increased by 1.48-fold with each standard deviation increase in soil Pb content [100]. Jameil found that occurrence of PE was associated with a significant increase in maternal Pb levels [101], which may include vasoconstriction and placental ischemia by inhibiting ATP activity and increasing circulating levels of endothelin [102,103]. Pregnant dental workers exposed to Hg have a higher incidence of PE [104].

## 3. Perspective

As a common and serious complication in pregnant women, PE poses a serious threat to the health of both mother and infant. Current treatments are mainly symptomatic, including hypotension, diuresis, and rational dilation, and do not address the underlying cause. As the pathological mechanism is unknown, there are no good predictive or intervention measures, and only termination of the pregnancy can relieve the short-term risk of the disease to the mother. Therefore, understand the risk factors and pathogenesis of PE may enable prevention and intervention. Although scientists have begun to recognise that exposure to xenobiotics during pregnancy is a risk factor for PE, the current study is limited. Most studies are based on in vitro cell culture experiments, lack of animal experimental tests or epidemiological studies based on human population. Besides, without clear adverse outcome pathways, is not easy to conduct risk assessment and management of these compounds. In future research, we should conduct more epidemiological investigations to clarify the causal relationship between exogenous compounds exposure and occurrence of PE. Studies on the adverse outcome pathways of PE induced by exogenous contaminants using in vitro models should be enhanced. Organoids can be used to study the effects and mechanisms of exogenous compounds on the developmental function of trophoblast cells. At the same time, omics, bioinformatics, and molecular biology can be used to help to understand the environmental etiology of PE. Research on the mixture toxicity effects of exogenous compounds on PE should be strengthened. We should also strengthen research on the combined effect of exogenous compounds and maternal nutrition intake or health status. 

## Data Availability

Not applicable.
